# Round the Hospitals

**Published:** 1919-04-12

**Authors:** 


### ROUND THE HOSPITALS.

The King has been pleased to award the Royal Red Cross, First Class, to the following ladies in recognition of valuable nursing services under the British Red Cross Society, or Order of St. John of Jerusalem in England, rendered in connection with the war:—Miss E. T. Bickerton, Matron, Prince of Wales' General Hospital; Mrs. F. Eadie, Matron, East Lancashire Red Cross Hospital, Didsbury College, Manchester; Miss C. F. Grasett, Matron, Auxiliary Hospital for Officers, 19 Hyde Park Gardens, London; Miss M. I. Jones, Matron, Auxiliary Military Hospital, Park House, Market Harborough, Leicestershire; Mrs. J. L. Latter, Matron, Red Cross Hospital, Knighton, Radnorshire; Mrs. J. G. Laurie, Matron, Red Cross Hospital for Officers, 28 Marlborough Buildings, Bath;

**ROUND THE HOSPITALS—*(continued).***

Miss R. E. M. MacWatters, Matron, Almondsbury Subsidiary Hospital, Bristol; Mrs. S. E. O'Keeffe, Commandant and Matron, Baldwin Auxiliary Hospital, Griffithtown, Monmouthshire; Mrs. M. Peiniger, Matron, Syon Auxiliary Hospital, Brentford; Mrs. M. Shaw, Commandant and Lady Superintendent Nurse, Spring Hall Auxiliary Military Hospital, Halifax, Yorkshire; Mrs. E. M. Wisdom, Matron, The Suffolk Hospital, Ampton Hall, Bury St. Edmunds. A very long list of awards to the Second Class of the Order, which we regret we have not space to print, will be found in the *Times* of the 5th inst.

At the Investiture held in the Ball-room of Buckingham Palace at 11 A.M. on April 3, the King personally conferred the Order of the Royal Red Cross on the following ladies: First class—Sister Anna Weatherstone, Q.A.I.M.N.S. (R.); and Matron Mabel Tisdale, T.F.N.S. Second class—Sister Mary Aitken, Sister Ruby Cameron, and Sister Lucy Deakin, Q.A.I.M.N.S. (R.); Matron Alice Wellstead, Sister Helen Callan, Sister Margaret Cox, Sister Helen Fergusson, Sister Lucy Rangescroft, and Staff Nurse Ethel Philps, T.F.N.S.; Matron Amy Moffat, Civil N.S.; Matron Alice Lovell, Sister Caroline Parker, Sister Florence Piza, and Sister Molly Ruddock, B.R.C.S.; Sister Gertrude Doherty and Sister Sadie MacDonald, Australian A.N.S.; Mrs. Jean Duka, V.A.D. These ladies had the honour of being received at Marlborough House by Queen Alexandra, subsequent to the Investiture.

At the second Investiture last week, held at Buckingham Palace on April 5, the King personally conferred the Order of the Royal Red Cross, Second Class, on the following ladies:—Sister Katharine Duff, late Q.A.I.M.N.S. (R.); Sister Mary Walker, T.F.N.S.; and Sister Agnes Gallop, Canadian A.N.S. The King conferred the Military Medal for bravery in the field on Matron Edith Campbell, Canadian A.N.S. All were subsequently received at Marlborough House by Queen Alexandra.

The Standing Committee of the House of Commons, to which the Nurses' Registration Bill prepared by the Central Committee has been referred, held its first sitting on April 8. It consists of fifty-five members, whose names are given on page 49, and is charged with the duty of examining, criticising, and amending the Bill. It is well known that the promoters of the College Bill, while approving wholeheartedly the principle of the Bill to give State recognition to nurses, are not in accord with the Central Committee's proposals for carrying Registration into effect. Their main objections centre round the manner in which the General Nursing Council, which will have very extensive powers, is to be elected; and that in which the Provisional Nursing Council is to be constituted. If the Bill became law in the form in which it was introduced nurses would be in a permanent minority on the General Nursing Council which is to control their profession, since only eighteen members of the Council out of forty-one would be elected as "direct representatives of the women nurses in the General Register." Moreover, on the Provisional Council, which will have unique powers in the interval before the General Nursing Council can be elected, the College of Nursing, representing 13,000 trained nurses, is accorded only two representatives, while ten are accorded to the little societies, the number of whose members has never yet been divulged, affiliated to the Central Committee.

On behalf of the College of Nursing, Colonel Raw, at the meeting on the 8th, moved two amendments to the present Bill removing these defects. His first amendment (see page 49) was ruled out of order, two previous amendments which were accepted having increased the number of the General Nursing Council to forty-five, of whom a majority (twenty-four) will be nurses. As regards the Provisional Council, the College of Nursing amendment proposed that it should consist of thirty-six persons. Three representatives are assigned to the British Medical Association, three to the Medico-Psychological Association, three to the Association of Poor Law Unions, and three to the British Hospitals Association. The remaining twenty-four seats are to be divided equally between the Central Committee for the State Registration of Nurses and the Council of the College of Nursing. The amendment also limits the functions of this Provisional Council and prescribes that it shall give way to the elected Council so soon as the number of registered nurses numbers 20,000. Major Barnett opposed the amendment, which was withdrawn in the hope that before the next meeting of the Standing Committee (fixed for 10th inst.) some agreement may be come to as to the constitution of the Council. There is grave danger to the profession in giving this initial unelected body powers to draw up all the rules under which the nursing profession is to be permanently governed. It would undoubtedly be far better to delay the passage of this long-anticipated reform rather than run any risk of placing nurses under the irresponsible yoke of persons not elected by nurses themselves for this office. A report of the proceedings of the Standing Committee appears on page 49.

Great changes are taking place in the organisation of the Nursing Department at the Middlesex Hospital. A new scale of salaries has come into force under which the sisters receive a substantial augmentation of pay. The salary of the massage sister-in-charge will be £100, rising to £150; that of the night superintendent, and also of the electrical department sister, £70-£80; home sisters, £65-£80; sister-midwife £60-£80; sister out-patient department, £55-£75; ward sisters £50-£70; staff nurses, £40. Probationers are to begin at £16. The fourth year's training is no longer compulsory, but certificated nurses from this and other recognised hospitals and infirmaries will be granted facilities for training during a fourth year

**ROUND THE HOSPITALS—(*continued*).**

in special subjects. The working hours are to be reduced to fifty-six a week, with off-duty times extended, and to enable this necessary reform to be carried out additional nurses are to be engaged. The acuteness of the problems involved in bettering the lot of hospital nurses is illustrated by the fact that the alterations we have outlined will cost the Middlesex £1,518 for the first year and more later on.

The post of tutor sister to the London Hospital which, as we announced last week, has been created, has been filled by the appointment of Miss Emily Frances Saunders. Miss Saunders trained at the London, having entered there in 1905. For eleven years she held the post of assistant sister in the receiving-room, and more recently that of head class sister. She left the service of the hospital a year ago and has been lecturing on bacteriology to district nurses in Lincolnshire. Miss Saunders matriculated at the London University, and has won distinctions in English and literature. Her work at the London has not yet been definitely arranged, but we understand one of her chief duties will be to give individual teaching to those probationers who have missed lectures and instruction classes through illness or other causes.

We welcome with much pleasure the announcement that, on the initiative of the matron, Miss Coggins, the Board of Management of the Royal Albert Edward Infirmary, Wigan, has adopted a scheme whereby the nurses will have one full day in seven off duty. It will become operative as soon as the necessary arrangements can be made. It has not been found practicable to arrange for the sisters to have this privilege, but in lieu of it they have been granted two weeks' holiday to be taken in winter, in addition to the annual month's holiday taken in the summer. This is quite a novel arrangement, and we believe it will be highly appreciated. The need for a break in the middle of winter is acutely felt by the heads of nursing departments, and we trust the matron is also to enjoy this refreshment. The salaries at the Royal Albert Edward Infirmary are now as follows:—Probationers, £16, £20, £24; staff nurses, £40, £42, £44; ward sisters, £50, rising by £5 increment to £60. Other grades of the nursing staff have received advances in their salaries. The matron invites application from suitable ladies as probationers to meet the increase in staff rendered necessary by the changes.

There appears to be a great dearth of nurses in North Wales. At the annual meeting of the North Wales Nursing Association, held at Colwyn Bay, with the Lord Lieutenant of Carnarvonshire in the chair, reference was made to the difficulties experienced in keeping the staff up to strength. Doubtless the subscribers would cordially assent to such an increase in salaries as would seem to be indicated by this scarcity.

It is with pleasure we announce that the Nation's Fund for Nurses has offered to raise the sum of £7,000 a year for three years for the Council of Queen Victoria's Jubilee Institute for Nurses for the purpose of meeting the increased cost of the training, supply and supervision of the nurses, the reduction of fees from the local Nursing Associations in order to enable them to raise the salaries of their nurses, and for increasing the Queen's Nurses' Benefit Fund, which is used for the personal benefit of the Queen's nurses throughout the United Kingdom. The Council of Queen Victoria's Jubilee Institute for Nurses has accepted this offer, and this object will therefore be added to the aims of the Nation's Fund for Nurses, whose purpose is to help the various nursing institutions as well as the individual nurses themselves.

The pressing need for a Central Fund to relieve nurses in distress has been already very forcibly brought to the attention of the committee which is administering the Nation's Tribute Fund. One case is that of a military nursing sister suffering from mental breakdown due to the long-continued strain of her Army duties. She was in danger of removal to a pauper ward in the asylum where she was under care, but owing to prompt action on the part of the committee was spared this humiliation, and is now, after several months' relief from financial difficulties, so completely cured as to be able to receive the Royal Red Cross from the King at one of the Investitures at Buckingham Palace. The War Office has tardily granted her £100 a year until she is able to resume work. Weekly pensions sufficient to cover the cost of living are now being granted by the Nation's Tribute Fund to many old and infirm nurses in their own homes, and others are being maintained in sanatoria, nursing homes, and asylums. The case of an Army nursing sister who retired invalided after the South African War on the princely sum of 8d. a day, and is now crippled and helpless, is, we fear, by no means so exceptional as might be thought.

Ireland is doing exceedingly well with its tribute to nurses. It will be remembered that Sir Arthur Stanley pleaded in Dublin last year for the sum of £10,000 from "all Ireland." The task of collecting this amount was undertaken by an influential committee, and a Vice-President who will sit on the Council of the Nation's Tribute to Nurses has been appointed from every county to look after the interests of nurses within her sphere. Representatives from many nursing associations are to sit on the Executive Committee. The sum collected to date already exceeds the amount appealed for by over £200, and donations are still pouring in. All the money will be needed, and more.

We are glad to learn that the War Office has agreed to eliminate Category B from Army Council Instruction No. 65, referring to the Military

**ROUND THE HOSPITALS—(*continued*).**

Massage Service. The Incorporated Society of Trained Masseuses took a strong tone on the matter from the outset, and Sir Cooper Perry, who has done an immense deal for this Society and its members, was able to show incontrovertibly at a meeting specially convened for this purpose that there is an abundant supply of fully trained masseuses, so that the supposed necessity for filling posts with the partly trained does not exist. The result is an object-lesson in the advantages of organisation. The Society has also made a protest in regard to the personnel of the future Pensions' Massage Service.

---

The need of the moment is for the hospitals to grant facilities for nurses to obtain training in public health work. For this reason we welcome particularly the announcement that the Brompton Hospital for Consumption is inaugurating a special year's course, which not only ensures a thorough practical knowledge of tuberculosis nursing and preventive work in connection with the disease, but also combines with it facilities for obtaining the health visitor's certificate of the Sanitary Institute. Nurses who wish to take this valuable year's course must be qualified for it by three years' general work. and will enter at Brompton with the status of staff nurses at a salary of £40 a year. They will attend one course of fifteen lectures, in spring or autumn. They will get first-rate practical training in all the latest developments of tuberculosis nursing in the wards and out-patient departments, including the effects of the disease on the throat—a very important branch of the subject—while at the sanatorium experience in open-air treatment will be available. There will be qualifying examinations, and a certificate will be granted to those who pass, which, it is needless to say, will be of high value in the eyes of municipal authorities. Nurses who take this course will be encouraged to enter for the health visitor's course of the Sanitary Institute, and will be accorded time for attendance at these lectures and preparation for the examination.

---

We trust that matrons of provincial and other hospitals which may be unable, for lack of opportunity, to afford special tuberculosis training will bring the advantages thus offered at the Brompton Hospital for Consumption to the notice of any of their newly certificated nurses who may desire to qualify for this special work, in order that they may correspond with the matron, Miss Redl, and obtain full particulars of the course. It is being inaugurated by a series of fifteen free lectures, to be delivered at the hospital on Tuesdays and Fridays at 8 P.M. during April and May, to which outside nurses and others are invited. The first four lectures have already been held, and have attracted large and deeply interested audiences. The course constitutes the most remarkable opportunity of gratuitous post-graduate instruction recently offered to nurses. The lectures on the 15th, 25th, and 29th of this month are on "Pulmonary Consumption: Clinical," by Dr. T. Gwynne Maitland; "Surgical Tuberculosis: Clinical," by Dr. R. C. Wingfield; and "The X-ray Evidence," by Dr. Stanley Melville.

---

We very much like the letter of "Mary Hine," published in the *Times* of April 4. Its tone is admirable, and its reasoning irresistible. The Army authorities clearly halted between two opinions in settling the status of the Army nurses. They could not rise to the notion of conferring military titles. They stuck to the bestowal of the Military Medal in place of the Military Cross—a point which has wounded nursing sisters not a little. But in the matter of gratuities they put members of the Nursing Services on a level with officers, and in this they did right, for it was greatly to the nurses' advantage. No reasonable Army staff nurse or sister would exchange a gratuity of £7 10s. to £10 for each year's service for the month's pay on furlough granted to the men.

---

We doubt whether Army nurses will feel grateful to Mrs. Bart Kennedy for representing them in the columns of the *Times* as a destitute, homeless, and spendthrift class. To describe them as "kicked out" of the Service because, more than six months after the armistice, they have at last been set free in response to their own earnest request, backed by the vociferous call of the public, is extremely injudicious. Members of the military Nursing Services are not penniless, and object very strongly indeed to being held out to the public as objects of pity. Their main desire at the moment is summed up in the words "Save us from our friends." The College of Nursing, however, put the views of the nurses before the War Office, and the Army Nursing Board, though holding that the nurses have no real grievance, has decided that to ease the situation they shall in future be granted one week's notice with full pay and allowances. All or part of this week may be taken as leave.

---

The Inter-Allied Red Cross Conference opened at Cannes on April 1, and will extend over two weeks. Mr. Henry P. Davison, former President of the American Red Cross War Council, opened the proceedings. In addition to the medical and surgical experts from England, America, France, Italy, and Japan, it has been thought advisable to include representatives of the nursing profession, whose judgment of the future work for the sick best to be undertaken by voluntary workers will be indispensable. Miss Lloyd Still, matron of St. Thomas's Hospital, and Head of the Nightingale School, and Miss Gill, matron of the Royal Infirmary, Edinburgh, have placed their services at the disposal of the British Red Cross Society. Both these ladies are members of the Council of the College of Nursing.

